# Guidelines for dementia or Parkinson’s disease with depression or anxiety: a systematic review

**DOI:** 10.1186/s12883-016-0754-5

**Published:** 2016-11-25

**Authors:** Zahra Goodarzi, Bria Mele, Selynne Guo, Heather Hanson, Nathalie Jette, Scott Patten, Tamara Pringsheim, Jayna Holroyd-Leduc

**Affiliations:** 1 Department of Community Health Sciences, University of Calgary, Calgary, Canada; 2 Department of Medicine, University of Calgary and Alberta Health Services, Calgary, Canada; 3 Faculty of Medicine, Undergraduate Medical Education, University of Toronto, Toronto, Canada; 4 Seniors Health Strategic Clinical Network, Alberta Health Services, Alberta, Canada; 5 Department of Clinical Neurosciences, University of Calgary, Calgary, Canada; 6 Hotchkiss Brain Institute, and O’Brien Institute for Public Health, University of Calgary and Alberta Health Services, Calgary, Canada; 7 Department of Psychiatry and Pediatrics, University of Calgary and Alberta Health Services, Calgary, Canada; 8 Department of Psychiatry, University of Calgary and Alberta Health Services, Calgary, Canada; 9 Mathison Centre for Mental Health Research and Education, University of Calgary, Calgary, Canada; 10 #1104-South Tower. Foothills Medical Centre 3301 Hospital Drive, Calgary, NW T2N 2T9 Canada

**Keywords:** Parkinson’s Disease, Dementia, Depression, Anxiety, Guidelines

## Abstract

**Background:**

Depression and anxiety remain under-diagnosed and under-treated in those with neurologic diseases such as dementia or Parkinson’s Disease (PD). Our objectives were to first, to provide a synthesis of high quality guidelines available for the identification and management of depression or anxiety in those with dementia or PD. Second, to identify areas for improvement for future guidelines.

**Methods:**

We searched MEDLINE, PsycINFO, and EMBASE (2009 to July 24, 2015), grey literature (83 sources; July 24-Sept 6, 2015), and bibliographies of included studies. Included studies were evaluated for quality by four independent reviewers the AGREE II tool. Guideline characteristics, statements and recommendations relevant to depression or anxiety for dementia and PD were then extracted. (PROSPERO CRD: 42016014584)

**Results:**

8121 citations were reviewed with 31 full text articles included for assessment with the AGREE II tool. 17 were of sufficient quality for inclusion. Mean overall quality scores were between 4.25 to 6.5. Domain scores were lowest in the areas of stakeholder involvement, applicability, and editorial independence.

Recommendations for the screening and diagnosis of depression were found for PD and dementia. There was little evidence to guide diagnosis or management of anxiety. Non-pharmacologic therapies were recommended for dementia patients. Most advocated pharmacologic treatment for depression, for both PD and dementia, but did not specify an agent due to lack of evidence.

**Conclusions:**

The available recent high quality guidelines outline several recommendations for the management of comorbid depression or anxiety in PD or dementia. However there remain significant gaps in the evidence.

**Electronic supplementary material:**

The online version of this article (doi:10.1186/s12883-016-0754-5) contains supplementary material, which is available to authorized users.

## Background

Persons experiencing neurologic disorders, such as dementia or Parkinson’s disease (PD), and depressive or anxiety disorders have poorer outcomes with reduced quality of life, poor functional status and worsened cognition [1–8].

It is estimated that the prevalence of depression in dementia is approximately 25% with anxiety occurring in up to 75% [7, 9–11]. In PD, approximately 17% of patients experience major depression and anxiety between 3.6 to 40% [2, 12].

Despite awareness of these comorbidities, depression and anxiety remain under-diagnosed and under-treated in those with neurologic diseases [1, 3, 13–17]. Only 20% of PD patients diagnosed with depression receive therapy [18]. This represents a significant knowledge-to-practice gap. One way to address this is through the use of Clinical Practice Guidelines (CPGs) [19]. CPGs synthesize available evidence based on a systematic review of the literature, clinical expertise and patient preferences [19]. CPGs are targeted at practitioners who apply the recommendations to clinical decision-making and reduce disparities in care [19–22].

Thus, in the setting of PD and dementia, CPGs should enable the appropriate management of depression and anxiety [23–26]. Despite available CPGs, these disorders remain under-managed, suggesting these CPGs are underused or lack sufficient recommendations [26–28]. Multiple available guidelines of varied quality leads to uncertainty as to which CPGs should be used in practice. Our primary aim is to synthesize the high-quality evidence-based CPGs available for diagnosis, and management of depression or anxiety in those with dementia or PD. We chose to summarize and evaluate guidelines as the majority of physicians will use CPGs as a tool to review evidence and inform practice. Secondarily we aim to, identify areas gaps within the existing guidelines to inform future guideline development. This provides a broad over view of evidence in the area and identifies areas for further study and development.

## Methods

The study protocol follows the recommendations provided by the Preferred Reporting Items for Systematic Reviews and Meta-Analyses (PRISMA)—Protocols Statement [29] and guidelines and the protocol was registered with PROSPERO [30] (CRD: 42016014584).

### Search strategy

The literature search was developed in conjunction with an experienced librarian (DL) and was verified independently by a second librarian (HLR), using the Peer Review of Electronic Search Strategies (PRESS) methodology [31]. Any recommendations were incorporated into the final search.

Databases included MEDLINE, EMBASE, and PsycINFO. Clusters of terms (controlled vocabulary and key words) were used to search each database; these include dementia, Parkinson’s disease, depression, anxiety and CPGs (Additional file 1: Box S1). The search was completed by cluster, first searching the terms in each cluster (combined with the Boolean operator ‘OR’) and keyword searches of abstracts and titles. The clusters were then combined with ‘AND’. We searched for several pathological variants of dementia including Alzheimer’s disease, vascular, frontotemporal, Lewy Body disease, Huntington’s Disease, CADASIL, primary progressive aphasia, and Creutzfeldt Jakob (Additional file 1: Box S1). We included relevant derivatives of terms or broad key words related to depressive or anxiety disorders (Additional file 1: Box S1).

This was augmented by a search of the grey literature (Additional file 2: Table S1). This search was limited from 2009 to search date, such that we would only capture CPGs developed within the past 5 years; given the evidence that CPGs may become out of date after only 3 years [32]. All languages were included in this search.

### Selection & eligibility

All citations were reviewed for eligibility by two independent authors; citations meeting initial eligibility criteria were included in full text review. If there was disagreement at the abstract stage, the full article was pulled for review. Bibliographies for all included articles were searched. If multiple CPGs were identified from a single agency on the same topic the most recent was used.

At the first stage of abstract review, any article that represented a guideline for PD or dementia was included. Eligibility at the full text stage required that the CPGs included at least one recommendation related to depression and/or anxiety in patients with PD and/or dementia. The kappa statistic was used to quantify inter-rater reliability.

For non-English articles that met eligibility at the full text stage, the language was determined using online translation software. Citations were translated using the online (Google translate) function to determine if an article was a guideline. When included, the documents were searched using translated relevant terms; for example, if a guideline pertained to PD in the abstract, the text was searched for depression or anxiety (and all translated synonyms). If those criteria were met, the full guideline was translated and reviewed.

### Assessment of quality

The Appraisal of Guidelines Research & Evaluation (AGREE II) tool was used to assess guideline quality [33]. This tool was designed to evaluate guideline quality and to aid in guideline development and reporting [33]. The tool includes 6 domains covering scope and purpose, stakeholder involvement, rigour of development, clarity of presentation, applicability and editorial independence [33]. Within each domain there are between 2 to 8 questions, to a total of 23 [33]. Each item is rated from 1 (not included or very poorly reported) to 7 (exceptional reporting of all criteria outlined in the AGREE II Manual) [33].

Each domain was scored independently by four reviewers, along with the assignment of an overall score. An initial assessment of 5 citations was done and compared across all 4 reviewers [33]. The 4 reviewers met to discuss discrepancies and address questions about rating, before the remainder of the guidelines were reviewed and scored. This also served to ensure that all raters were aligned in their understanding of the AGREE II items. Any further discrepancies were resolved by discussion.

Domain scores pooled across the 4 assessors were calculated, as outlined in the AGREE II user manual [33]. The higher score indicates a higher quality across rated items. It has been demonstrated that the quality across the AGREE II domains predicts guideline implementation [33]. The mean overall quality scores with standard deviations (SD) were calculated, as well as for each domain item. CPGs with a mean overall quality score 5 or greater were assigned at least moderate quality and included in further analysis. CPGs with a score below 3 were excluded due to low quality. A score less than 5 but greater than 3 were re-evaluated and inclusion status was decided by consensus.

### Data extraction & synthesis of evidence

Guideline characteristics were extracted by one author (ZG) and independently verified by a second author (BM). Items extracted included the primary conditions covered, region/organizations, number of committee members, numbers of references, and sources of funding.

Two independent reviewers then extracted relevant recommendations (ZG, BM). Specifically, guidelines were searched for any mention of relevant recommendations and supporting text or statements. Three authors reviewed the extracted recommendations (ZG, BM and JHL). Recommendations were compiled across the guidelines into relevant categories and subcategories, and reported using descriptive statistics including the quality, number of guidelines supporting the statement and subpopulations included. As the evidence in the guidelines is represented by practice recommendations, it was not amenable to meta-analysis. The main output of this systematic review was an appraisal of the quality of all guidelines pertaining to comorbid depression or anxiety in PD or dementia, and a synthesis of the recommendations across the different guidelines. Data were analyzed using STATA 13.1 (Stata Corp. College Station, TX).

## Results

### Study selection

**Fig. 1:**
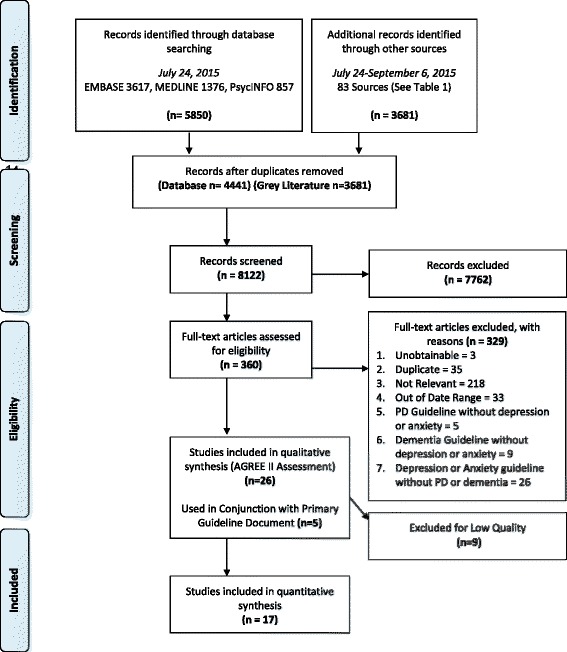
PRISMA Flow Diagram

The database search generated 4441 citations after duplicates were removed, with a further 3681 citations identified from the grey literature (Fig. 1). When screened for eligibility, 360 citations met criteria for full text review (κ = 0.88, 95.7% agreement). At this stage most articles were excluded because they were not relevant (*n* = 218), were not guidelines, or were unrelated guidelines. Other common reasons for exclusion at the full text stage were being out of the date range (*n* = 33) or a duplicate (*n* = 35). Excluded citations also included 26 mental health guidelines that did not address PD or dementia. Similarly there were 5 PD and 9 dementia guidelines that did not address depression or anxiety. The dementia guidelines primarily pertained to Alzheimer’s disease, vascular dementia, general dementia care and one referred to Lewy Body Disease. Of these articles, 4 were identified to be summary documents of included guidelines and were used as supplemental material to these included guidelines. Twenty-six CPGs met all eligibility criteria and were evaluated using the AGREE II tool, of which 17 met the quality cut off for inclusion.

### Guideline characteristics

The 17 included guidelines addressed PD (*n* = 5), dementia (*n* = 8) and mental health (*n* = 4) CPGs (Table 1). They included recommendations from many regions, including Canada (*n* = 2), USA (*n* = 3), Pan-European (*n* = 4), UK (*n* = 2), Scotland (*n* = 1), Spain (*n* = 2), South Korea (*n* = 1) and international (*n* = 2). The associations or organizations are outlined in Table 1. All guidelines used a method for grading the evidence (Additional file 3: Figure S1). Most guidelines were funded through government or non-commercial funding; only two CPGs had some pharmaceutical funding.

**Table 1:** Guideline Characteristics

Author (year)	Organiz-ation	Primary condition^a^	Focus	Region of origin	# of Committee members^i^	#of Refs	Systematic search (Y/N)	Grading of evidence (Y/N)	Funding (NS, P, NC, G)^j^	Mean quality score
Zesiewicz et al*.* (2010) [35]	The American Academy of Neurology (AAN)	PD	Treatment	USA	9	40	Y	Y	NC	4.5
No Author (2010)^b^ [37]	Scottish Intercollegiate Guidelines Network (SIGN)	PD	Diagnosis Treatment	Scotland	20	189	Y	Y	G	6
Grimes et al*.* (2012)^c^ [34]	Canadian Neurological Sciences Federation (CNSF) & Parkinson Society Canada	PD	Diagnosis Treatment	Canada	22	62	Y	Y	NC & P	6.5
Berardelli et al*.* (2013) [38]	European Federation of Neurological Societies & Movement Disorder Society—European Section (EFNS-MDS-ES)	PD	Diagnosis	Europe	25	245	Y	Y	NS ^h^	5
Ferreira et al*.* (2013) [40]	European Federation of Neurological Societies & Movement Disorder Society—European Section (EFNS-MDS-ES)	PD	Treatment	Europe	22	363	Y	Y	NC	4.5
Hort et al*.* (2010) [47]	European Federation of Neurological Societies (EFNS)	Dementia	Diagnosis Treatment	Europe	8	100	Y	Y	NC	4.25
No Author (2010) [42]	Ministry of Health, Social Services and Equality & Agency for Health Quality and Assessment of Catalonia (AIAQS)	Dementia	Diagnosis Treatment	Spain	67	688	Y	Y	NC & G	5.75
No Author (2011)^d^ [41]	National Institute for Health and Care Excellence, National Collaborating Centre for Mental Health, British Psychological Society & The Royal College of Psychiatrists (NICE)	Dementia	Diagnosis & Treatment	UK	28	NN^h^	Y	Y	NC & G	6.5
Ihl et al*.* (2011) [44]	World Federation of Societies of Biological Psychiatry (WFSBP)	Dementia	Treatment	International	39	215^f^	Y	Y	NC	4.5
No Author (2011) [43]	Clinical Research Centre for Dementia (CRCD)	Dementia	Diagnosis	South Korea	20	NN^h^	Y	Y	G	5.25
O’Brien et al*.* (2011) [60]	British Association of Psychopharmacology (BPA)	Dementia	Treatment	UK	16	148^f^	N	Y	NC & P	4
Sorbi et al*.* (2012) [45]	European Federation of Neurological Societies & European Neurological Society (EFNS-ES)	Dementia	Diagnosis Treatment	Europe	17	189	Y	Y	NC	4.5
Gauthier et al (2012)^e^ [50]	Canadian Consensus Conference on the Diagnosis and Treatment of Dementia (CCCDTD4)	Dementia	Diagnosis Treatment	Canada	38	19	Y	Y	NC	5.5
Gelenberg et al*.* (2010)^g^ [39]	American Psychiatric Association (APA)	Depression	Treatment	USA	7	1170	Y	Y	NC	4.75
Dua et al*.* (2011) [49]	World Health Organization (WHO)	Mental Health	Diagnosis Treatment	International	29	36	Y	Y	NC & G	5.5
No Author (2012) [46]	Ministry of Health, Social Services and Equality & Galician Health Technology Assessment Agency (Availia-T)	Suicide	Diagnosis Treatment	Spain	24	683	Y	Y	NC & G	5
Mitchell et al*.* (2013) [48]	Institute for Clinical Systems Improvement (ICSI)	Depression	Diagnosis Treatment	USA	14	331	Y	Y	NC	5.75

^a^ Dementia guidelines primarily included Alzheimer’s disease, vascular dementia, general dementia care and one referred to Lewy Body Disease

^b^ Includes Grosset et al*.* [54]

^c^ Includes Patel et al*.* [61]

^d^ Originally created in 2007 and updated in 2011

^e^ Includes Moore et al*.* [62], Herrman et al*.* [63]

^f^ Number counted from the text

^g^ Includes Recommendations Referenced in Rabin et al*.* [64]

^h^ NS: Not Stated, NN: Not Numbered

^i^ Committee members—extracted from paper as listed (e.g. authors listed, guideline development/working groups etc.)

^j^ NC: Non-Commercial, G: Government, Pharmaceutical, NS: Not Stated

References: The American Academy of Neurology (AAN) [35], Scottish Intercollegiate Guidelines Network (SIGN) [37, 54], Canadian Neurological Sciences Federation (CNSF) [34], Parkinson’s Society Canada [34], European Federation of Neurological Societies (EFNS) (*n* = 4) [38, 40, 45, 47], Movement Disorders Society-European Section (MDS-ES) [38, 40], National Institute for Health and Care Excellence (NICE) [41], Ministry of Health, Social Services and Equality & Agency for Health Quality and Assessment of Catalonia (AIAQS) [42], British Psychological Society [41], The Royal College of Psychiatrists [41], World Federation of Societies of Biological Psychiatry (WFSBP) [44], Clinical Research Centre for Dementia (CRCD), British Association of Psychopharmacology (BPA) [60], European Neurological Society, Canadian Consensus Conference on the Diagnosis and Treatment of Dementia (CCCDTD4) [50], American Psychiatric Association (APA) [39], World Health Organization (WHO) [49], Ministry of Health, Social Services and Equality & Galician Health Technology Assessment Agency (Availia-T) [46] and the Institute for Clinical Systems Improvement (ICSI) [48]

### Study quality

These 26 CPGs were assessed for quality using all 23 items across the 6 domains of the AGREE II tool. Nine guidelines were excluded for low quality. Six were excluded with an overall mean rating ranging from 2.25 to 3.75. Three had ratings of 4–4.5, where decision to exclude was by consensus. A low rating was typically due to unclear methods; thus scoring low on rigour of development, applicability and editorial independence. Authors of guidelines were contacted for more information in the case that an item was unclear and responses were incorporated in the quality assessment.

The 17 included guidelines had mean overall scores from 4 to 6.5 (Table 2). When examining the individual domain scores, the highest rated domain was Domain 4: Clarity of Presentation (mean score 77.0; SD 11.4). This was followed by Domain 1: Scope and Purpose (mean score 72.1; SD 12.1). Domain 5: Applicability was the lowest rated domain (mean score 41.5; SD 22.6). Stakeholder involvement (Domain 2) also had a low score (mean score 54.5; SD 23.3).

**Table 2:** Domain scores from AGREE II evaluation

Guideline (year)	Domain 1 score scope & purpose	Domain 2 score stakeholder involvement	Domain 3 score rigour of development	Domain 4 score clarity of presentation	Domain 5 score applicability	Domain 6 score editorial independence
Parkinson’s Disease						
Zesiewicz et al. (2010) [35]	56.9	29.2	64.6	72.2	17.7	79.2
SIGN (2010)^a^ [37]	80.6	80.6	72.9	91.7	72.9	22.9
Grimes et al*.* (2012)^c^ [34]	70.8	95.8	90.6	87.5	60.4	58.3
Berardelli et al. (2013) [38]	72.2	19.4	47.9	86.1	12.5	6.3
Ferreira et al*.* (2013) [40]	47.2	15.3	43.2	66.7	6.25	20.8
Dementia						
NICE (2011)^b^ [41]	83.3	81.9	86.5	87.5	64.6	47.9
Hort et al*.* (2010) [47]	58.3	38.9	54.2	66.7	25.0	62.5
AIAQS (2010) [42]	87.5	69.4	73.4	84.7	57.3	79.2
Ihl et al*.* (2011) [44]	68.1	38.9	57.8	48.6	19.8	64.6
CRCD (2011) [43]	86.1	62.5	74.5	81.9	51.0	54.2
O’Brien et al (2011) [60]	59.7	63.9	46.4	76.4	20.8	68.8
Sorbi et al*.* (2012) [45]	68.1	38.9	53.7	65.3	26.0	62.5
Gauthier et al (2012)^d^ [50]	73.6	70.8	70.8	87.5	50.0	79.2
Mental Health						
Gelenberg et al*.* (2010) [39]	68.1	41.7	61.5	66.7	32.3	60.4
Dua et al*.* (2011) [49]	70.8	41.7	66.7	84.7	68.7	93.8
Avalia-T (2012) [46]	88.9	70.8	79.2	75.0	49.0	60.4
Mitchell et al*.* (2013) [48]	86.1	66.7	75.0	80.6	71.9	85.4
Average Domain Score (SD)	72.1 (12.1)	54.5 (23.3)	65.8 (13.9)	77.0 (11.4)	41.5 (22.6)	59.2 (23.7)

*SD* Standard Deviation

^a^ Includes Grosset et al. [54]

^b^ Originally created in 2007 and updated in 2011

^c^ Includes Patel et al. [61]

^d^ Includes Moore et al. [62], Herrman et al. [63]

The mean rating across each question in the domain scores were also examined to explore differences between domains (Additional file 4: Table S2). Question one pertaining to the overall objectives was the highest rated item at 5.88 (SD 0.61), followed by link between evidence and recommendations at 5.78 (SD 0.51). The lowest rated item was providing a procedure for updating the guideline is provided, with a mean rating of 3.16 (SD 1.73). The views and preferences of the target population have been sought was also rated poorly with a mean score of 3.25 (SD 1.92). All items in Domain 5 had low mean scores, ranging between 3.27 (SD 1.46) for resource implications and 3.72 (SD 1.53) for advice on putting recommendations into practice.

### Guideline recommendations

The details of extracted recommendations are summarized in the Table 3 for PD and Table 4 for dementia. 21 categories of recommendations were extracted in total.

**Table 3:** Statements & recommendations for Parkinson’s disease

Anxiety	
Evidence for the Management & Treatment of Anxiety in PD is Lacking.	
Level of Evidence	AAN Level U (Uncertain or Lack of Evidence)
Guidelines	Zesiewicz et al. (2010) [35], Grimes et al. (2012) [34]
Depression	
Screening for Depression in PD is recommended.	
Level of Evidence	EFNS Level A (Effective), SIGN Grade C (Case Control to Cohort Evidence)
Guidelines	Berardelli et al*.* (2013) [38], Grosset et al*.* (2010) [54]
There are several available tools screening for Depression in PD.	
Level of Evidence	SIGN Level C & Good Practice Point (GDS, BDI, HADS, MADRS & HDRS) & EFNS Class I (Diagnostic Accuracy Study)(MDS-UPDRS)
Guidelines	Grosset et al. (2010) [54], Berardelli et al*.* (2013) [38]
Comment	A patient with PD should be screened for depression with either a clinician or self-rated tool. Diagnosis should not be based on the solely on the tool. Those with a positive screening test should be referred for further assessment and diagnosis (including collateral history).
Practitioners should have a low threshold for diagnosing Depression in PD.	
Level of Evidence	CFNS Good Practice Point
Guidelines	Grimes et al*.* (2012) [34]
Treatment of Depression in PD needs to be individualized to each case.	
Level of Evidence	CFNS Good Practice Point
Guidelines	Grimes et al*.* (2012) [34]
Anti-depressant Therapy is recommended; there is little evidence to suggest one agent over another.	
Guidelines	Gelenberg et al*.* (2010) [39], Grosset et al*.* (2010) [54]
Tricyclic Antidepressants (e.g. Amitriptyline or Desipramine) have some evidence for treatment, but this must be balanced with the adverse effects (e.g. Anticholinergic).	
Level of Evidence	CFNS Level C (Possibly Effective)
Guidelines	Grimes et al*.* (2012) [34], Grosset et al. (2010) [54], Gelenberg et al. (2010) [39]
Selective Serotonin Reuptake Inhibitors have some evidence for treatment, but this must be balanced with the adverse effects (e.g. RLS, PLM, RBD).	
Level of Evidence	EFNS Class II (Prospective Matched Group Cohort or Controlled Trial) to Class IV (Uncontrolled Studies), APA Level II (Moderate Clinical Evidence)
Guidelines	Ferreira et al. (2013) [40], Gelenberg et al. (2010) [39]
Certain agents such as Amoxapine or Lithium should be avoided due to worsening of PD Symptoms.	
Guidelines	Gelenberg et al. (2010) [39]
There is some evidence for the use of dopamine agonists (e.g. Pramipexole) & MAOI (e.g. Selegiline) for depression, but not for levodopa.	
Level of Evidence	EFNS Class I (RCT), Class III (Other Controlled Trial), APA Level I (Recommended with substantial confidence)
Guidelines	Ferreira et al. (2013) [40], Gelenberg et al. (2010) [39], Grimes et al. (2012) [34]
There is insufficient evidence regarding the use of ECT, TCMS and psychotherapy in depression with PD.	
Guidelines	Ferreira et al. (2013) [40], Gelenberg et al. (2010) [39], Grimes et al. (2012) [34]

**Table 4:** Statements & recommendations for Dementia

Anxiety	
Patients with Dementia should be assessed for Anxiety (e.g. HADS).	
Level of Evidence	AIAQS Level D (Expert Opinion)
Guidelines	AIAQS (2010) [42], NICE (2011) [41]
Psychological Interventions can be considered for Anxiety in Dementia	
Guidelines	NICE (2011) [41]
There is little evidence about the treatment of Anxiety in those with Dementia. Cholinesterase Inhibitors can be considered for treating Dementia-related behaviours, including anxiety.	
Level of Evidence	AIAQS Level A (Meta-analysis or RCT)
Guidelines	AIAQS (2010) [42]
Depression	
Patients experiencing Dementia should be evaluated for Depression, including possible secondary causes.	
Level of Evidence	CRCD Level A (Useful), AIAQS Level D, WFSBP Grade 3 (Limited Evidence from Controlled Studies), EFNS GPP
Guidelines	NICE (2011) [41], AIAQS (2010) [42], CRCD (2011) [43], Sorbi et al (2012) [45], Ihl et al. (2011) [44]
Patients with Depression in Dementia should be evaluated for suicide risk, however evidence varies.	
Level of Evidence	APA Level I (Substantial Clinical Confidence) or Inconclusive
Guidelines	Gelenberg et al. (2010) [39], Avalia-T (2012) [46]
Use of a valid screening tool (e.g. CSDD, GDS, HADS or DMAS) for Depression is recommended.	
Level of Evidence	AIAQS Level D to Good Practice Point, Low Quality Evidence, EFNS GPP/Class II (Prospective Study)
Guidelines	Gelenberg et al. (2010) [39], AIAQS (2010) [42], Sorbi et al (2012) [45], Hort et al (2010) [47], Mitchell et al. (2013) [48]
fMRI needs further study to determine its utility in Depression in the context of Dementia	
Level of Evidence	CCCDT4 Grade 2C (Moderate Recommendation, Low Level Evidence)
Guidelines	Gauthier et al. (2012) [50]
Therapy for Depression in Dementia should include a variety of Non-pharmacologic options.	
Level of Evidence	AIAQS Level C (Case-control, Cohort), APA Level II (Moderate Clinical Confidence)
Guidelines	NICE (2011) [41], AIAQS (2010) [42], Gelenberg et al. (2010) [39], Mitchell et al. (2013) [48]
Comment	These include: cognitive behavioural therapy, reminiscence therapy, multi-sensory stimulation, animal-assisted therapy, exercise, stimulation-oriented treatment (recreational or pleasurable activities), or improvements to a living situation. Consider the involvement of carers.
Although evidence is mixed, a trial of Anti-depressants could be considered for Depression in Dementia.	
Level of Evidence	CCCDT4 Grade 2A (Moderate Recommendation, High Level Evidence), EFNS Class IV (Un-blinded, Expert Opinion), WFSBP Grade 5 (Inconsistent Results), APA Level II (Moderate Clinical Confidence)
Guidelines	Gauthier et al. (2012) [50], NICE (2011) [41], Sorbi et al (2012) [45], Gelenberg et al. (2010) [39], Ihl et al. (2011) [44], Dua et al (2011) [49]
When choosing an anti-depressant (E.g. SSRIs, SNRIs or TCAs) it is important to consider the anticholinergic side effects.	
Level of Evidence	EFNS Level B (Case-control, Cohort), EFNS Class IV (Un-blinded, Expert Opinion), APA Level I (Substantial Clinical Confidence) to APA Level II (Moderate Clinical Confidence), AIAQS Level B
Guidelines	Gauthier et al. (2012) [50], NICE (2011) [41], Sorbi et al (2012) [45], Hort et al (2010) [47], Gelenberg et al. (2010) [39], AIAQS (2010) [42]
Comment	SSRIs (Citalopram or Sertraline) and TCAs have similar efficacy, but TCAs are not recommended given anticholinergic effects. SSRIs appear to be better tolerated. Other agents such as bupropion, venlafaxine and mirtazapine may be effective.
Stimulants can be considered for treatment of Depression in Dementia.	
Level of Evidence	APA Level III (Depends on Individual Circumstances), AIAQS Level B (Case-control, Cohort)
Guidelines	Gelenberg et al. (2010) [39], AIAQS (2010) [42]
Cholinesterase Inhibitors can be considered for treating Dementia-related behaviours, including depression.	
Level of Evidence	AIAQS Level A (Meta-analysis or RCT)
Guidelines	AIAQS (2010) [42]
ECT can be considered in certain cases for Depression in those with Dementia.	
Level of Evidence	APA Level II (Moderate Clinical Confidence)
Guidelines	Gelenberg et al. (2010) [39]
Cholinesterase Inhibitors may improve neuropsychiatric symptoms in Lewy Body Disease	
Level of Evidence	Level A (Meta-analysis or RCT)
Guidelines	O’Brien et al (2011) [60]

### Parkinson’s disease recommendations

Only two guidelines discussed anxiety in those with PD [34, 35]. These stated there was little evidence for either the diagnosis or treatment of anxiety in PD, and that there was insufficient evidence for the treatment of anxiety with levodopa [34, 35].

There were clear recommendations surrounding the diagnosis of depression in PD [34, 37, 38]. Clinicians should have a low threshold for the diagnosis of depression in PD given the difficulties making a diagnosis [34]. Use of a validated tool for detecting depression (or neuropsychiatric symptoms) was advocated by two guidelines, with varying levels of recommendations [37, 38]. Tools that were recommended include the HDRS, the MADRS or the UPDRS—Part 1 Non-Motor, among others [37, 38]. The diagnosis should be made based on a clinical interview and not based on the tool alone and should seek collateral information from carers [37].

Antidepressant therapy is recommended, however there is little evidence to support one agent over another (*n* = 2) [37, 39]. Additionally, the choice of an agent must be individualized (*n* = 1) and the practitioner should consider side effects and drug interactions prior to initiation [34]. There have been prior studies on the tricyclic antidepressants (TCAs), specifically amitriptyline, and although they were beneficial for mood, this was offset by the side effects (*n* = 3) [34, 37, 39]. One guideline noted that selective serotonin reuptake inhibitors (SSRIs) showed some benefit in uncontrolled studies [39, 40], but noted that the SSRIs could worsen PD symptoms of restless legs (RLS), periodic limb movement (PLM) and REM sleep behaviour disorder (RBD) (*n* = 2) [39, 40]. It is recommended to avoid amoxapine and lithium in those with PD, due to the risk of worsening motor symptoms (*n* = 1) [39].

There is some weak evidence supporting the use of dopamine agonists and monoamine oxidase inhibitors for the management of depression in PD (*n* = 3) [34, 39, 40]. Pramipexole was suggested to have an antidepressant effect not solely due a motor effect [40]. Selegiline has some antidepressant effects but further studies are needed [39]. If the mood symptoms are only present during off periods, it was suggested that patients might benefit from drugs addressing the motor symptoms [34]. However there was no evidence levodopa alone affected mood [40].

Other therapies for depression are not well explored in PD. The European Federation of Neurological Sciences (EFNS) concluded there was insufficient data to recommend psychotherapy, electroconvulsive therapy (ECT) or transcranial magnetic stimulation (TCMS) [40]. Other guideline assert that ECT has been used in PD, but that there are no specific trials in PD and is associated with risk (*n* = 2) [34, 39].

### Dementia recommendations

It is recommended that patients with dementia be assessed for anxiety (*n* = 2), however there is no clear consensus on what tools to use [41, 42]. One guideline recommended the use of the Hospital Anxiety Depression Scale [42]. The evidence for the treatment of anxiety in dementia is lacking (*n* = 1) [42].

It is recommended that patients with dementia be evaluated and re-evaluated over time for depression (*n* = 5) [41–45]. As part of this assessment, patients should be evaluated for other secondary causes of depression. It is suggested that these patients be assessed for suicidality by one guideline [39], however another reported there was inconclusive evidence regarding this [46].

The use of a valid screening tool was recommended for depression case finding (*n* = 5) in dementia, including the CSDD, GDS or Dementia Mood Assessment Scale (DMAS) [39, 42, 45, 47, 48]. The CSDD was more commonly recommended given it is a clinician-rating tool that involves caregivers with higher sensitivity (*n* = 4) [39, 45, 47, 48].

Therapy for depression in those with dementia should include a variety of non-pharmacologic options (*n* = 4) such as stimulation oriented, cognitive behavioural, reminiscence, exercise or multi-sensory therapy [39, 41, 42, 48]. Pharmacologic therapy is recommended despite variable evidence (*n* = 6) [41, 42, 44, 45, 49, 50]. It is suggested by one guideline that, if there is no improvement with non-pharmacologic therapy, an antidepressant be considered [50]. Another notes that for moderate-severe depression, pharmacologic treatment is warranted (*n* = 1) [49]. However, there needs to be a clear risk-benefit assessment and discussion (*n* = 1) [41]. Based largely on clinical experience, most guidelines recommend the use of SSRIs given the lower side effect profile over TCAs (*n* = 6) [39, 41, 42, 45, 49, 50]. The concern with TCAs is largely anticholinergic side effects causing worsened cognition [42, 50]. Other antidepressants such as mirtazapine, bupropion, and venlafaxine may also be of benefit (*n* = 1) [42]. Other adjunct therapies recommended include stimulants (*n* = 2) [39, 42], cholinesterase inhibitors (*n* = 1) [42] and ECT on a case-by-case basis (*n* = 1) [39].

## Discussion

This study provides a synthesis and quality assessment of available guidelines for the management of depression or anxiety in PD or dementia. We identified clear gaps in guideline quality and the evidence, which inform future research and knowledge translation.

### Guideline quality

Guidelines that were excluded due to low quality were typically those that lacked explicit development methods, thus ratings across all the domains were low. When examining the AGREE II ratings overall, the lowest rating was in assessing the guideline description of barriers and facilitators, implementation, resource implications, or monitoring/auditing criteria (Domain 5). In fact, few guidelines had discrete sections addressing knowledge translation. The concern about guideline applicability was explored in a 2015 systematic review [51], which found that applicability scored lower than any other domain [51, 52]. If guidelines rarely address their implementation in practice, then there will be continued practice variation. There is clear evidence supporting the use of implementation tools to improve guideline uptake [51]. Thus making guidelines without a clear knowledge translation plan does a disservice to stakeholders [51].

The engagement of patients and caregivers was notably absent in CPG development. This process is important, as it is aimed at improving implementability, by ensuring the recommendations are comprehensive, adaptable and applicable to the target group and have an open process [53]. Given the constant changing nature of evidence, having up-to-date guidelines certainly makes a difference to the validity [32]. However, the lowest rated item was for the guideline update procedures.

### Guideline content

There is an overall lack of recommendations related to the diagnosis or treatment of anxiety in either PD or dementia. This stems from the fact there is little evidence on how to approach the assessment. One guideline suggested the use Hospital Anxiety and Depression Scale for dementia, but they did not provide diagnostic accuracy information or suggestions for implementation [42]. There is also a concern that the medications traditionally used for anxiety can have major adverse effects [35], and there are few studies to guide treatment. Anxiety was less frequently mentioned than depression in the included CPGs, and in some cases was only mentioned in combination with other neuropsychiatric symptoms. The overall lack of evidence for anxiety care in PD and dementia is a major gap in the current research.

Guidance for depression was present in a higher proportion of guidelines. Despite this, there is variability in the reporting of levels of evidence and recommendations (Additional file 3: Figure S1). In some cases the recommendations for depression in PD only had 1 or 2 guidelines supporting them, indicating variance in guideline reporting. In other cases recommendations were vague, which can lead to difficulty with end user interpretation and implementation [36].

It is clear that screening for depression with a validated tool in PD is recommended, although evidence varies [37, 38]. It is recommended, as a good practice point, that any diagnosis of depression is not made solely on a brief assessment tool, as these tools are more focused on case finding [37]. Although this is an important concept in detection, it was only recommended by one guideline [54]. A 2015 systematic review identified several validated tools for the detection of depression in PD, with the GDS-15 having the highest pooled sensitivity (0.81; 95% CI 0.64, 0.91) and area under the curve (0.94) [55].

Recommendations surrounding non-pharmacologic therapy were few, stating there was insufficient evidence for the use of psychotherapy, ECT or TMS [34, 39, 40]. Two recent trials demonstrated the effectiveness of cognitive behavioural therapy in PD [56, 57]. This highlights the need for further large high quality studies on a range of non-pharmacologic therapies and the need for constant update of guidelines. Pharmacological therapy is recommended for managing depression in PD, but there is little evidence on choosing agents [39, 54]. This has resulted in a variety of treatment recommendations, with little evidence to direct clinical practice.

Depression in dementia was more frequently addressed. However, these recommendations also had varied guideline and evidentiary support. Guidelines supported the evaluation of depression in dementia, but evidence ranged from high quality to good practice points [41–45]. Commonly recommended tools were the CSDD and GDS, with preference towards the CSDD due to better accuracy [39, 42, 45, 47, 48]. This was confirmed by a 2015 systematic review of depression tools for dementia, finding that the CSDD had a area under the curve of 0.89 [58].

Interestingly, the issue of evaluating for suicide risk was raised in two guidelines with divergent recommendations [39, 46]. One stating there was inconclusive evidence [46] and another stating substantial evidence [39]. It is unclear why there is such a difference in reported evidence; perhaps development groups have different evidence available or differing interpretations of the evidence.

There are stronger recommendations for non-pharmacologic treatment in dementia than in PD, outlining several options [41, 42, 45, 47, 48]. The evidence for pharmacologic therapy is described as mixed with Grade 2A (Moderate Recommendation, High Level Evidence) to Class IV (Un-blinded Study, Expert Opinion) [39, 41, 44, 45, 49, 50]. Again SSRIs and TCAs are the focus, with TCAs being less likely to be recommended due to side effects [39, 42, 45, 47, 50]. For those with dementia, there were more options recommended for therapy including stimulants, cholinesterase inhibitors and ECT [39, 42].

#### Limitations

There is a well-recognized issue with heterogeneity in the terms used to refer to guidelines [52]. For our database search we used indexed terms from each of the three databases as well as key words using known nomenclature for guidelines and the comorbidities. It is also possible that the addition of the depression or anxiety criteria to the search may have been restrictive, however without these terms the search was impractical. To address this, we developed the search strategy with experts in the area of guideline systematic review and an experienced librarian, and we had an external reviewer independently assess the search strategy. To reduce the risk of missing literature not indexed in databases we contacted experts, searched references of included studies and performed an extensive search of the grey literature search.

## Conclusions

Given the burden of comorbid mental illness in dementia and PD, it is key that we understand clearly the current knowledge base so we can improve care for these populations. This study provides a synthesis and quality assessment of the relevant guidelines. By synthesizing the recommendations, we identified areas of knowledge that are potentially ready to be translated into practice but also clear evidence gaps. This data was further evaluated in a subsequent study by stakeholders in focus groups to understand the other barriers and facilitators to the use of guidelines. This was to inform and help develop a comprehensive knowledge/end-user focused plan for addressing these gaps.

## Additional files

Additional file 1: Box S1. Search Strategy. (DOCX 22 kb)
Additional file 2: Table S1. Grey Literature Sources (*n* = 83). (DOCX 15 kb)
Additional file 3: Figure S1. Evidence Levels & Grading Schemes Used Across Guidelines [34–49, 59]. (DOCX 65.4 kb)
Additional file 4: Table S2. Mean Domain Question Scores From AGREE II Evaluation. (DOCX 16 kb)

## Abbreviations

AAN: American Academy of Neurology
APA: American Psychiatric Association
AIAQS: Agency for Health Quality and Assessment of Catalonia
Avalia-T: Galician Health Technology Assessment Agency
BDI: Beck Depression Inventory
BPA: British Association of Psychopharmacology
CADASIL: Cerebral Autosomal-Dominant Arteriopathy with Subcortical Infarcts and Leukoencephalopathy
CCCDTD4: Canadian Consensus Conference on the Diagnosis and Treatment of Dementia
CNSF: Canadian Neurological Sciences Foundation
CPG: Clinical Practice Guideline
CRCD: Clinical Research Centre for Dementia
CSDD: Cornell Scale for Depression in Dementia
DMAS: Dementia Mood Assessment Scale
ECT: Electroconvulsive Therapy
EFNS: European Federation of Neuroscience
GDS: Geriatric Depression Scale
GRADE: Grading of Recommendations Assessment, Development and Evaluation
HADS: Hospital Anxiety and Depression Scale
HDRS: Hamilton Depression Rating Scale
ICSI: Institute for Clinical Systems Improvement
MADRS: Montgomery Åsberg Depression Rating Scale
MDS: Movement Disorders Society
NICE: National Institute of Clinical Excellence
PD: Parkinson’s Disease
PLM: Periodic Limb Movement Syndrome
PRESS: Peer Review of Electronic Search Strategies
PRISMA: Preferred Reporting Items for Systematic Reviews and Meta-Analyses
RBD: REM Sleep Behaviour Disorder
RCT: Randomized Control Trial
REM: Rapid Eye Movement
RLS: Restless Legs Syndrome
SD: Standard Deviation
SIGN: Scottish Intercollegiate Guidelines Network
SSRI: Selective Serotonin Reuptake Inhibitor
TCA: Tricyclic Acid Antidepressants
TCMS: Transcranial Magnetic Stimulation
UPDRS: Unified Parkinson’s Disease Rating Scale
WFBSP: World Federation of Societies of Behavioural Psychiatry
WHO: World Health Organization

## References

[1] Pachana NA, Egan SJ, Laidlaw K, Dissanayaka N, Byrne GJ, Brockman S, Marsh R, Starkstein S. Clinical issues in the treatment of anxiety and depression in older adults with Parkinson’s disease. Movement disorders: official journal of the Movement Disorder Society. 2013;28(14):1930–4. 10.1002/mds.25689 24123116

[2] Reijnders JS, Ehrt U, Weber WE, Aarsland D, Leentjens AF (2008). A systematic review of prevalence studies of depression in Parkinson’s disease. Mov Disord.

[3] Schrag A, Leentjens AF (2012). Parkinson disease: scales to detect depression in Parkinson disease. Nat Rev Neurol.

[4] Weintraub D, Moberg PJ, Duda JE, Katz IR, Stern MB (2004). Effect of psychiatric and other nonmotor symptoms on disability in Parkinson’s disease. J Am Geriatr Soc.

[5] Kostic VS, Pekmezovic T, Tomic A, Jecmenica-Lukic M, Stojkovic T, Spica V, Svetel M, Stefanova E, Petrovic I, Dzoljic E (2010). Suicide and suicidal ideation in Parkinson’s disease. J Neurol Sci.

[6] Hughes TA, Ross HF, Mindham RH, Spokes EG (2004). Mortality in Parkinson’s disease and its association with dementia and depression. Acta Neurol Scand.

[7] Orgeta V, Qazi A, Spector AE, Orrell M (2014). Psychological treatments for depression and anxiety in dementia and mild cognitive impairment. Cochrane Database Syst Rev.

[8] Banerjee S, Hellier J, Dewey M, Romeo R, Ballard C, Baldwin R, Bentham P, Fox C, Holmes C, Katona C (2011). Sertraline or mirtazapine for depression in dementia (HTA-SADD): a randomised, multicentre, double-blind, placebo-controlled trial. Lancet.

[9] Enache D, Winblad B, Aarsland D (2011). Depression in dementia: epidemiology, mechanisms, and treatment. Curr Opin Psychiatry.

[10] Riley RJ, Burgener S, Buckwalter KC (2014). Anxiety and stigma in dementia: a threat to aging in place. Nurs Clin North Am.

[11] Van der Mussele S, Bekelaar K, Le Bastard N, Vermeiren Y, Saerens J, Somers N, Marien P, Goeman J, De Deyn PP, Engelborghs S (2013). Prevalence and associated behavioral symptoms of depression in mild cognitive impairment and dementia due to Alzheimer’s disease. Int J Geriatr Psychiatry.

[12] Dissanayaka NN, Sellbach A, Matheson S, O’Sullivan JD, Silburn PA, Byrne GJ, Marsh R, Mellick GD (2010). Anxiety disorders in Parkinson’s disease: prevalence and risk factors. Mov Disord.

[13] Marsh L (2013). Depression and Parkinson’s disease: current knowledge. Curr Neurol Neurosci Rep.

[14] Djamshidian A, Friedman JH (2014). Anxiety and depression in Parkinson’s disease. Curr Treat Options Neurol.

[15] Knapskog AB, Barca ML, Engedal K (2011). A comparison of the validity of the Cornell Scale and the MADRS in detecting depression among memory clinic patients. Dement Geriatr Cogn Disord.

[16] Modrego PJ, Ferrandez J (2004). Depression in patients with mild cognitive impairment increases the risk of developing dementia of Alzheimer type: a prospective cohort study. Arch Neurol.

[17] Ownby RL, Crocco E, Acevedo A, John V, Loewenstein D (2006). Depression and risk for Alzheimer disease: systematic review, meta-analysis, and metaregression analysis. Arch Gen Psychiatry.

[18] Frisina PG, Borod JC, Foldi NS, Tenenbaum HR (2008). Depression in Parkinson’s disease: health risks, etiology, and treatment options. Neuropsychiatr Dis Treat.

[19] Davis D, J. Goldman, P. Valerie: Handbook on Clinical Practice Guidelines. In*.*: Canadian Medical Association; 2007. 10.1503/cmaj.070880 PMC2043082 17984472

[20] Gagliardi AR, Brouwers MC, Palda VA, Lemieux-Charles L, Grimshaw JM. How can we improve guideline use? A conceptual framework of implementability. Implementation Sci. 2011;6:26. https://www.ncbi.nlm.nih.gov/pubmed/21426574 10.1186/1748-5908-6-26 PMC3072935 21426574

[21] Graham R, Mancher M, Miller-Wolman D, Greenfield S, Steinberg EH (2011). Clinical Practice Guidelines We Can Trust.

[22] Grimshaw JM, Thomas RE, MacLennan G, Fraser C, Ramsay CR, Vale L, Whitty P, Eccles MP, Matowe L, Shirran L (2004). Effectiveness and efficiency of guideline dissemination and implementation strategies. Health Technol Assess.

[23] Ramasubbu R, Taylor VH, Samaan Z, Sockalingham S, Li M, Patten S, Rodin G, Schaffer A, Beaulieu S, McIntyre RS (2012). The Canadian Network for Mood and Anxiety Treatments (CANMAT) task force recommendations for the management of patients with mood disorders and select comorbid medical conditions. Ann Clin Psychiatry.

[24] Ramasubbu R, Beaulieu S, Taylor VH, Schaffer A, McIntyre RS, Canadian Network for M, Anxiety Treatments Task F (2012). The CANMAT task force recommendations for the management of patients with mood disorders and comorbid medical conditions: diagnostic, assessment, and treatment principles. Ann Clin Psychiatry.

[25] Kerr MP, Mensah S, Besag F, de Toffol B, Ettinger A, Kanemoto K, Kanner A, Kemp S, Krishnamoorthy E, LaFrance WC (2011). International consensus clinical practice statements for the treatment of neuropsychiatric conditions associated with epilepsy. Epilepsia.

[26] Wilcock J, Iliffe S, Turner S, Bryans M, O’Carroll R, Keady J, Levin E, Downs M (2009). Concordance with clinical practice guidelines for dementia in general practice. Aging Ment Health.

[27] Salter K, McClure JA, Mahon H, Foley N, Teasell R (2012). Adherence to Canadian best practice recommendations for stroke care: assessment and management of poststroke depression in an Ontario rehabilitation facility. Top Stroke Rehabil.

[28] McCluskey A, Vratsistas-Curto A, Schurr K (2013). Barriers and enablers to implementing multiple stroke guideline recommendations: a qualitative study. BMC Health Serv Res.

[29] Moher D, Liberati A, Tetzlaff J, Altman DG, Group P. Preferred reporting items for systematic reviews and meta-analyses: the PRISMA statement. Bmj 2009, 339:b2535. 10.1136/bmj.b2535 PMC2714657 19622551

[30] Making the Case for Investing in Mental Health in Canada. In*.*: Mental Health Commission of Canada; 2013.

[31] PRESS: peer review of electronic search strategies. 2015 Guideline Explanation and Elaboration (PRESS E&E). Ottawa: Canadian Agency for Drugs and Technologies in Health; 2016. https://www.cadth.ca/sites/default/files/pdf/CP0015_PRESS_Update_Report_2016.pdf

[32] Martinez Garcia L, Sanabria AJ, Garcia Alvarez E, Trujillo-Martin MM, Etxeandia-Ikobaltzeta I, Kotzeva A, Rigau D, Louro-Gonzalez A, Barajas-Nava L, Diaz Del Campo P (2014). The validity of recommendations from clinical guidelines: a survival analysis. CMAJ.

[33] Brouwers MC, Kho ME, Browman GP, Burgers JS, Cluzeau F, Feder G, Fervers B, Graham ID, Grimshaw J, Hanna SE (2010). AGREE II: advancing guideline development, reporting and evaluation in health care. CMAJ.

[34] Grimes D, Gordon J, Snelgrove B, Lim-Carter I, Fon E, Martin W, Wieler M, Suchowersky O, Rajput A, Lafontaine AL (2012). Canadian guidelines on Parkinson’s disease. Can J Neurol Sci.

[35] Zesiewicz TA, Sullivan KL, Arnulf I, Chaudhuri KR, Morgan JC, Gronseth GS, Miyasaki J, Iverson DJ, Weiner WJ, Quality Standards Subcommittee of the American Academy of N (2010). Practice parameter: treatment of nonmotor symptoms of Parkinson disease: report of the quality standards subcommittee of the American Academy of Neurology. Neurology.

[36] Michie S, Johnston M (2004). Changing clinical behaviour by making guidelines specific. Bmj.

[37] Diagnosis and pharmacological management of Parkinson’s disease: A national clinical guideline. Scottish Intercollegiate Guidelines Network; 2010.

[38] Berardelli A, Wenning GK, Antonini A, Berg D, Bloem BR, Bonifati V, Brooks D, Burn DJ, Colosimo C, Fanciulli A, et al. EFNS/MDS-ES/ENS [corrected] recommendations for the diagnosis of Parkinson’s disease. European journal of neurology: the official journal of the European Federation of Neurological Societies. 2013;20(1):16–34. 10.1111/ene.12022 23279440

[39] Gelenberg AJ, Freeman MP, Markowitz JC, Rosenbaum JF, Thase ME, Trivedi MH, Van Rhoads RS. Practice Guideline for Treatment of Patients with Major Depressive Disorder 3rd Edition. American Pyschiatric Association; 2010.

[40] Ferreira JJ, Katzenschlager R, Bloem BR, Bonuccelli U, Burn D, Deuschl G, Dietrichs E, Fabbrini G, Friedman A, Kanovsky P, et al. Summary of the recommendations of the EFNS/MDS-ES review on therapeutic management of Parkinson’s disease. Eur J Neurol. 2013;20(1):5–15. https://www.ncbi.nlm.nih.gov/pubmed/23279439 10.1111/j.1468-1331.2012.03866.x 23279439

[41] Dementia: Guidelines on Supporting People with Dementia and their carers in Health and Social Care. National Institute for Health and Clinical Excellence. National Collaborating Centre for Mental Health. Social Care Institute for Excellence National Institute for Health and Clinical Excellence. In*.* London: The British Psychological Society & Gaskell The Royal College of Psychiatrists; 2011.

[42] Development Group of the Clinical Practice Guideline on the comprehensive care of people with Alzheimer’s disease and other dementias. Clinical Practice Guideline on the comprehensive care of people with Alzheimer’s disease and other dementias. *Quality Plan for the National Health System of the Ministry of Health, Social Policies and Equality Agència d’Informació, Avaluació i Qualitat en Salut of Catalonia Clinical Practice Guidelines in the Spanish National Health Service: AIAQS No 2009/07* 2010. http://www.guiasalud.es/GPC/GPC_484_Alzheimer_AIAQS_comp_eng.pdf

[43] Clinical Research Center for Dementia of South Korea. Clinical practice guideline for dementia. Part I: diagnosis & evaluation. Seoul: Clinical Research Center for Dementia of South Korea; 2011. p. 117. The guideline cited is available here: http://jkma.org/src/SM/jkma-54-861-s002.pdf

[44] Ihl R, Frolich L, Winblad B, Schneider L, Burns A, Moller HJ, Disease WTFoTGfAs, other D. World Federation of Societies of Biological Psychiatry (WFSBP) guidelines for the biological treatment of Alzheimer’s disease and other dementias. World J Biol Psychiatry. 2011;12(1):2–32. https://www.ncbi.nlm.nih.gov/pubmed/21288069 10.3109/15622975.2010.538083 21288069

[45] Sorbi S, Hort J, Erkinjuntti T, Fladby T, Gainotti G, Gurvit H, Nacmias B, Pasquier F, Popescu BO, Rektorova I, et al. EFNS-ENS Guidelines on the diagnosis and management of disorders associated with dementia. Eur J Neurol. 2012;19(9):1159–79. https://www.ncbi.nlm.nih.gov/pubmed/22891773 10.1111/j.1468-1331.2012.03784.x 22891773

[46] Working Group of the Clinical Practice Guideline for the Prevention and Treatment [trunc]. Clinical practice guideline for the prevention and treatment of suicidal behaviour. Madrid: Ministry of Health and Social Policy, Galician Health Technology Assessment Agency; 2012. p. 382. http://www.guiasalud.es/GPC/GPC_481_Conducta_Suicida_Avaliat_compl_en.pdf

[47] Hort J, O’Brien JT, Gainotti G, Pirttila T, Popescu BO, Rektorova I, Sorbi S, Scheltens P, Dementia ESPo (2010). EFNS guidelines for the diagnosis and management of Alzheimer’s disease. Eur J Neurol.

[48] Mitchell J, Trangle M, Degnan B, Gabert T, Haight B, Kessler D, Mack N, Mallen E, Novak H, Rossmiller D, et al. Adult Depression in Primary Care. Institute for Clinical Systems Improvement; 2013.

[49] Dua T, Barbui C, Clark N, Fleischmann A, Poznyak V, van Ommeren M, Yasamy MT, Ayuso-Mateos JL, Birbeck GL, Drummond C, et al. Evidence-based guidelines for mental, neurological, and substance use disorders in low- and middle-income countries: summary of WHO recommendations. PLoS Med. 2011;8(11):e1001122. https://www.ncbi.nlm.nih.gov/pubmed/22110406 10.1371/journal.pmed.1001122 PMC3217030 22110406

[50] Gauthier S, Patterson C, Chertkow H, Gordon M, Herrmann N, Rockwood K, Rosa-Neto P, Soucy JP (2012). Recommendations of the 4th Canadian consensus conference on the diagnosis and treatment of dementia (CCCDTD4). Can Geriatr J.

[51] Gagliardi AR, Brouwers MC (2015). Do guidelines offer implementation advice to target users? A systematic review of guideline applicability. BMJ Open.

[52] Sauro KM, Wiebe S, Dunkley C, Janszky J, Kumlien E, Moshe S, Nakasato N, Pedley TA, Perucca E, Senties H (2016). The current state of epilepsy guidelines: A systematic review. Epilepsia.

[53] Eccles MP, Grimshaw JM, Shekelle P, Schunemann HJ, Woolf S (2012). Developing clinical practice guidelines: target audiences, identifying topics for guidelines, guideline group composition and functioning and conflicts of interest. Implementation Sci.

[54] Grosset DG, Macphee GJ, Nairn M, Guideline Development G (2010). Diagnosis and pharmacological management of Parkinson’s disease: summary of SIGN guidelines. BMJ.

[55] Goodarzi ZS, Mrklas K, Roberts DJ, Jette N, Pringsheim T, Holroyd-Leduc J. Depression case finding in Parkinson’s disease patients: a systematic review of depression screening tools. Montreal: Canadian Geriatric Society Scientific Meeting 2015; 2015.

[56] Dobkin RD, Menza M, Allen LA, Gara MA, Mark MH, Tiu J, Bienfait KL, Friedman J (2011). Cognitive-behavioral therapy for depression in Parkinson’s disease: a randomized, controlled trial. Am J Psychiatry.

[57] Troeung L, Egan SJ, Gasson N (2014). A waitlist-controlled trial of group cognitive behavioural therapy for depression and anxiety in Parkinson’s disease. BMC Psychiatry.

[58] Goodarzi Z, Mele B, Roberts D, Holroyd-Leduc J (2015). Depression case finding in dementia patients: a systematic review of depression screening tools.

[59] Guidance notes for registering a systematic review protocol with PROSPERO. In: *PROSPERO: International prospective register of systematic reviews* Centre for Reviews and Dissemination, National Institute for Health Research; 2013.

[60] O’Brien JT, Burns A, Group BAPDC. Clinical practice with anti-dementia drugs: a revised (second) consensus statement from the British Association for Psychopharmacology. J Psychopharmacol. 2011;25(8):997–1019. https://www.ncbi.nlm.nih.gov/pubmed/21088041 10.1177/0269881110387547 21088041

[61] Patel T, Chang F, Parkinson Society C (2014). Parkinson’s disease guidelines for pharmacists. Can Pharm J.

[62] Moore A, Patterson C, Lee L, Vedel I, Bergman H, Canadian Consensus Conference on the D, Treatment of D (2014). Fourth Canadian Consensus Conference on the Diagnosis and Treatment of Dementia: recommendations for family physicians. Can Fam Physician.

[63] Herrmann N, Lanctot KL, Hogan DB. Pharmacological recommendations for the symptomatic treatment of dementia: the Canadian Consensus Conference on the Diagnosis and Treatment of Dementia 2012. Alzheimer’s Res Ther. 2013;5 Suppl 1:S5. https://www.ncbi.nlm.nih.gov/pubmed/24565367 10.1186/alzrt201 PMC3980908 24565367

[64] Rabins PV, Blacker D, Rovner BW, Rummans T, Schneider LS, Tariot PN, Blass DM. Practice Guidelines for the Treatment of Patients With Alzheimer’s Disease and Other Dementias. American Pyschiatric Association; 2007. 10.1176/appi.focus.15106 PMC6519627 31997970

