# Supplementary material for: Guidelines for dementia or Parkinson’s disease with depression or anxiety: a systematic review
Source: BMC Neurol. 2016 Nov 25;16:244. doi: 10.1186/s12883-016-0754-5 (PMC5124305; doi:10.1186/s12883-016-0754-5)
Supplement: Additional file 1: Box S1. — Search Strategy. (DOCX 22 kb) [file 12883_2016_754_MOESM1_ESM.docx]

**Additional file 1: Database Search Strategy**

**MEDLINE**

Database: Ovid MEDLINE(R) In-Process & Other Non-Indexed Citations and Ovid MEDLINE(R) <1946 to Present>

Search Strategy:

------------------------------------------------------

1 "guideline*".ab,kw,ti. (215252)

2 "quality*".ab,kw,ti. (629529)

3 "standard* of care".ab,kw,ti. (26227)

4 "best clinical practice*".ab,kw,ti. (260)

5 "appropriate* of care".ab,kw,ti. (5038)

6 "RAND appropriate* method*".ab,kw,ti. (60)

7 "practice parameter*".ab,kw,ti. (933)

8 "performance improve*".ab,kw,ti. (4857)

9 "evidence based recommendation*".ab,kw,ti. (2513)

10 "performance indicator*".ab,kw,ti. (2173)

11 "guideline* for care".ab,kw,ti. (900)

12 "care pathway*".ab,kw,ti. (1984)

13 "position paper*".ab,kw,ti. (2386)

14 "position statement*".ab,kw,ti. (2589)

15 "health technology assessment*".ab,kw,ti. (2605)

16 Practice Guideline/ or Guideline/ or Guideline Adherence/ (49701)

17 Evidence-Based Medicine/ (58467)

18 Health Planning Guidelines/ (3946)

19 patient care management/ or comprehensive health care/ or critical pathways/ or "delivery of health care"/ or disease management/ or patient-centered care/ or physician's practice patterns/ (149817)

20 "quality of health care"/ or guideline adherence/ or quality assurance, health care/ or quality improvement/ or quality indicators, health care/ (144606)

21 1 or 2 or 3 or 4 or 5 or 6 or 7 or 8 or 9 or 10 or 11 or 12 or 13 or 14 or 15 or 16 or 17 or 18 or 19 or 20 (1086304)

22 "anxious*".ab,kw,ti. (12354)

23 "anxiet*".ab,kw,ti. (126885)

24 Anxiety/ (57207)

25 Anxiety Disorders/ (24675)

26 22 or 23 or 24 or 25 (155679)

27 "depressive*".ab,kw,ti. (79546)

28 depressed.ab,kw,ti. (82203)

29 "depression*".ab,kw,ti. (243896)

30 "mood disorder*".ab,kw,ti. (12207)

31 Depression/ (83554)

32 depressive disorder/ or depressive disorder, major/ or depressive disorder, treatment-resistant/ (81018)

33 27 or 28 or 29 or 30 or 31 or 32 (365749)

34 "parkinson*".ab,kw,ti. (83495)

35 "dementia*".ab,kw,ti. (75175)

36 "alzheimer*".ab,kw,ti. (102055)

37 "frontotemporal*".ab,kw,ti. (7403)

38 "lewy bod*".ab,kw,ti. (6601)

39 "huntington*".ab,kw,ti. (13638)

40 "primary progressive aphasia*".ab,kw,ti. (688)

41 "creutzfeld-jakob*".ab,kw,ti. (164)

42 dementia/ or alzheimer disease/ or aphasia, primary progressive/ or creutzfeldt-jakob syndrome/ or dementia, vascular/ or frontotemporal lobar degeneration/ or huntington disease/ or lewy body disease/ (123386)

43 Dementia, Multi-Infarct/ (1096)

44 Parkinson Disease/ (51020)

45 Parkinsonian Disorders/ (5553)

46 Mood Disorders/ (11517)

47 33 or 46 (371304)

48 26 or 47 (457105)

49 CADASIL/ (576)

50 Frontotemporal Dementia/ (1274)

51 34 or 35 or 36 or 37 or 38 or 39 or 40 or 41 or 42 or 43 or 44 or 45 or 49 or 50 (260073)

52 48 and 51 (19123)

53 21 and 52 (2374)

54 **limit 53 to yr="2009 -Current" (1376)**

***************************

**PsycINFO**

Database: PsycINFO <1806 to July Week 3 2015>

Search Strategy:

------------------------------------------------------

1 "guideline*".ab,kw,ti. (44333)

2 "quality*".ab,kw,ti. (163685)

3 "standard* of care".ab,kw,ti. (3609)

4 "best clinical practice*".ab,kw,ti. (94)

5 "appropriate* of care".ab,kw,ti. (1322)

6 "RAND appropriate* method*".ab,kw,ti. (3)

7 "practice parameter*".ab,kw,ti. (279)

8 "performance improve*".ab,kw,ti. (2751)

9 "evidence based recommendation*".ab,kw,ti. (381)

10 "performance indicator*".ab,kw,ti. (945)

11 "guideline* for care".ab,kw,ti. (169)

12 "care pathway*".ab,kw,ti. (435)

13 "position paper*".ab,kw,ti. (666)

14 "position statement*".ab,kw,ti. (510)

15 "health technology assessment*".ab,kw,ti. (213)

16 "anxious*".ab,kw,ti. (17072)

17 "anxiet*".ab,kw,ti. (147755)

18 "depressive*".ab,kw,ti. (79329)

19 depressed.ab,kw,ti. (40877)

20 "depression*".ab,kw,ti. (184377)

21 "mood disorder*".ab,kw,ti. (11680)

22 "parkinson*".ab,kw,ti. (24034)

23 "dementia*".ab,kw,ti. (47404)

24 "alzheimer*".ab,kw,ti. (43805)

25 "frontotemporal*".ab,kw,ti. (3959)

26 "lewy bod*".ab,kw,ti. (2674)

27 "huntington*".ab,kw,ti. (3709)

28 "primary progressive aphasia*".ab,kw,ti. (526)

29 "creutzfeld-jakob*".ab,kw,ti. (22)

30 1 or 2 or 3 or 4 or 5 or 6 or 7 or 8 or 9 or 10 or 11 or 12 or 13 or 14 or 15 (211714)

31 Treatment Guidelines/ (4907)

32 clinical practice/ (13977)

33 Evidence Based Practice/ (12366)

34 "Quality of Care"/ (9798)

35 30 or 31 or 32 or 33 or 34 (235115)

36 22 or 23 or 24 or 25 or 26 or 27 or 28 or 29 (94811)

37 Parkinson's Disease/ (15986)

38 Dementia/ (26011)

39 Alzheimer's Disease/ (35025)

40 Dementia with Lewy Bodies/ (1311)

41 Semantic Dementia/ (1141)

42 Parkinsonism/ (2478)

43 Presenile Dementia/ (276)

44 Picks Disease/ (256)

45 Vascular Dementia/ (1849)

46 37 or 38 or 39 or 40 or 41 or 42 or 43 or 44 or 45 (71696)

47 Creutzfeldt Jakob Syndrome/ (602)

48 36 or 46 or 47 (97137)

49 Major Depression/ or Atypical Depression/ or Treatment Resistant Depression/ or "Depression (Emotion)"/ or Recurrent Depression/ (117785)

50 18 or 19 or 20 or 21 or 49 (237253)

51 Generalized Anxiety Disorder/ or Anxiety Disorders/ or Anxiety/ or Anxiety Management/ (62827)

52 16 or 17 or 51 (159837)

53 50 or 52 (335914)

54 Huntingtons Disease/ (2483)

55 48 or 54 (97161)

56 53 and 55 (13242)

57 35 and 56 (1482)

58 limit 57 to yr="2009 -Current" (857)

***************************

**EMBASE**

Database: Embase <1980 to 2015 Week 29>

Search Strategy:

------------------------------------------------------

1 "guideline*".ab,kw,ti. (317904)

2 "quality*".ab,kw,ti. (852308)

3 "standard* of care".ab,kw,ti. (41608)

4 "best clinical practice*".ab,kw,ti. (390)

5 "appropriate* of care".ab,kw,ti. (6584)

6 "RAND appropriate* method*".ab,kw,ti. (107)

7 "practice parameter*".ab,kw,ti. (1257)

8 "performance improve*".ab,kw,ti. (5963)

9 "evidence based recommendation*".ab,kw,ti. (3377)

10 "performance indicator*".ab,kw,ti. (3035)

11 "guideline* for care".ab,kw,ti. (1239)

12 "care pathway*".ab,kw,ti. (3652)

13 "position paper*".ab,kw,ti. (2814)

14 "position statement*".ab,kw,ti. (3087)

15 "health technology assessment*".ab,kw,ti. (3450)

16 "anxious*".ab,kw,ti. (16375)

17 "anxiet*".ab,kw,ti. (173966)

18 Anxiety/ (130802)

19 Anxiety Disorders/ (39045)

20 "depressive*".ab,kw,ti. (103664)

21 depressed.ab,kw,ti. (91013)

22 "depression*".ab,kw,ti. (316883)

23 "mood disorder*".ab,kw,ti. (18761)

24 "parkinson*".ab,kw,ti. (111067)

25 "dementia*".ab,kw,ti. (105709)

26 "alzheimer*".ab,kw,ti. (135974)

27 "frontotemporal*".ab,kw,ti. (10625)

28 "lewy bod*".ab,kw,ti. (9141)

29 "huntington*".ab,kw,ti. (16759)

30 "primary progressive aphasia*".ab,kw,ti. (1143)

31 "creutzfeld-jakob*".ab,kw,ti. (259)

32 Parkinson disease/ (104178)

33 parkinsonism/ (19901)

34 Pick presenile dementia/ or frontal variant frontotemporal dementia/ or senile dementia/ or multiinfarct dementia/ or semantic dementia/ or presenile dementia/ or dementia/ or frontotemporal dementia/ (99336)

35 CADASIL/ (1447)

36 Alzheimer disease/ (136199)

37 Creutzfeldt Jakob disease/ (9099)

38 primary progressive aphasia/ (1084)

39 Lewy body/ (5621)

40 24 or 25 or 26 or 27 or 28 or 29 or 30 or 31 or 32 or 33 or 34 or 35 or 36 or 37 or 38 or 39 (372226)

41 anxiety neurosis/ or "mixed anxiety and depression"/ or generalized anxiety disorder/ (15351)

42 16 or 17 or 18 or 19 or 41 (234712)

43 depression assessment/ or late life depression/ or treatment resistant depression/ or organic depression/ or depression/ or endogenous depression/ or long term depression/ or atypical depression/ or agitated depression/ or masked depression/ or reactive depression/ or major depression/ (298773)

44 20 or 21 or 22 or 23 or 43 (503331)

45 practice guideline/ (258652)

46 clinical pathway/ (6594)

47 standard/ (341430)

48 clinical protocol/ (72395)

49 professional standard/ (30377)

50 evidence based practice/ or evidence based medicine/ (123150)

51 quality control/ or health care quality/ (316946)

52 1 or 2 or 3 or 4 or 5 or 6 or 7 or 8 or 9 or 10 or 11 or 12 or 13 or 14 or 15 or 45 or 46 or 47 or 48 or 49 or 50 or 51 (1832439)

53 42 or 44 (623380)

54 40 and 53 (37476)

55 52 and 54 (5642)

56 **limit 55 to yr="2009 -Current" (3617)**
