# Supplementary material for: Guidelines for dementia or Parkinson’s disease with depression or anxiety: a systematic review
Source: BMC Neurol. 2016 Nov 25;16:244. doi: 10.1186/s12883-016-0754-5 (PMC5124305; doi:10.1186/s12883-016-0754-5)
Supplement: Additional file 2: Table S1. — Grey Literature Sources (n = 83). (DOCX 15 kb) [file 12883_2016_754_MOESM2_ESM.docx]

| Table S1: Grey Literature Sources (n=83)  Search Dates: July 24-Sept 6, 2015 | |
| --- | --- |
| Disease Specific Websites for Dementia | Alzheimer’s Society (Canadian, UK, USA), Alzheimer’s Association, Alzheimer’s Australia, Alzheimer’s Disease International, Alzheimer Europe, Alzheimer’s Foundation of America, Alzheimer Scotland, Alzheimer’s Research UK, American Academy of Neurology, Australia’s Dementia Collaborative Research Centres, The Association for Frontotemporal Degeneration, Dementia Action Alliance, Dementia and Neurodegeneration, National Stroke Association, Lewy Body Dementia Association, Lewy Body Society, The Australian Fronto-temporal Dementia Association, Huntington’s Disease Association England & Wales, Australian Huntington’s Disease Association, Huntington Society of Canada, Huntington’s Disease Association, Huntington’s Disease Society of America, International Huntington Association, The UK CJD Surveillance Unit, Creutzfeldt-Jakob Disease Foundation, Creutzfeldt-Jakob Disease International Surveillance Network, CJD Support Group Network, National Aphasia Association, Young Dementia UK, CADASIL Foundation, Cure CADASIL.org |
| Disease Specific Websites for PD | Parkinson’s Society of Canada, Parkinson’s Clinical Guidelines, Pacific Parkinson’s Research Institute, Parkinson’s Disease Foundation, National Parkinson’s Foundation, Parkinson’s UK, American Parkinson Disease Association, Michael J Fox Foundation, Parkinson Alliance, Parkinson Action Network |
| Disease Specific Websites for Depression and Anxiety | Depression Alliance, Canadian Mental Health Association, Mood Disorders Society of Canada, DepressionHurts.ca, Mental Health America, Anxiety and Depression Association of America, Anxiety UK, Anxiety Canada |
| Guideline Specific Websites | Canadian Medical Association, National Guidelines Clearinghouse, US Centres for Disease Control and Prevention Task Force, NHS Evidence - National Library of Guidelines including NICE, National Clinical Guideline Centre UK, NHS Evidence in Health and Social Care, Canadian Task Force on the Preventative Health Care, New Zealand Guidelines Group, and Guide to Clinical Preventive Services, Scottish Intercollegiate Guideline Network (SIGN), BCguidelines.ca, European Federation of Neurological Societies, Movement Disorder Society, American Psychiatric Association, American Psychological Association, British Association for Counseling and Psychotherapy, British Psychological Society, National Institute for Health and Clinical Excellence, Canadian Psychiatric Association, World Psychiatric Association, Australian Psychological Society, The Royal Australian & New Zealand College of Psychiatrists, National Institute on Aging, National Collaborating Centre for Mental Health, National Institute of Neurological Communicative Disorders, US Preventive Services Task Force, American College of Physicians, American Academy of Family Physicians, American Association of Geriatric Psychiatry, Assessing Care for Vulnerable Elders |
| Conference Proceedings | World Parkinson’s Congress, Canadian Neurological Sciences Federation Meeting, American Academy of Neurology Meeting, Movement Disorder Society International Conference, International Congress of Parkinson’s Disease, World Congress of Neurology, American Geriatric Society, Canadian Conference on Dementia, Annual Dementia Conference, Alzheimer’s Neuroscience Conference, FTD Conference, UK Dementia Congress, World Congress of Neurology, International Conference on Alzheimer's and Parkinson's diseases, Canadian Neurological Sciences Federation Meeting. |
