# Supplementary material for: Guidelines for dementia or Parkinson’s disease with depression or anxiety: a systematic review
Source: BMC Neurol. 2016 Nov 25;16:244. doi: 10.1186/s12883-016-0754-5 (PMC5124305; doi:10.1186/s12883-016-0754-5)
Supplement: Additional file 3: Figure S1. — Evidence Levels & Grading Schemes Used Across Guidelines [34–49, 59]. (DOCX 65.4 kb) [file 12883_2016_754_MOESM3_ESM.docx]

|  |  | **Evidence Levels & Grading Schemes Used** | | | | | |
| --- | --- | --- | --- | --- | --- | --- | --- |
|  |  | **AAN** | **NICE** | **EFNS** | **GRADE** | **SIGN** | **Other** |
| **Author (Year) & Organization of Included Guidelines** | Zesiewicz *et al*. (2010) **AAN** |  |  |  |  |  |  |
|  | No Author (2010) **SIGN** |  |  |  |  |  |  |
|  | Grimes *et al.* (2012) **CNFS & PSC** |  |  |  |  |  |  |
|  | Berardelli *et al*. (2013) **EFNS-MDS-ES** |  |  |  |  |  |  |
|  | Ferreira *et al.* (2013) **EFNS-MDS-ES** |  |  |  |  |  |  |
|  | Hort *et al.* (2010) **EFNS** |  |  |  |  |  |  |
|  | No Author (2010) **AIQAS** |  |  |  |  |  |  |
|  | No Author (2011) **NICE** |  |  |  |  |  |  |
|  | Ihl *et al.* (2011) **WFSBP** |  |  |  |  |  |  |
|  | No Author (2011) **CRCD** |  |  |  |  |  |  |
|  | O'Brien *et al*. (2011) **BPA** |  |  |  |  |  |  |
|  | Sorbi *et al.* (2012) **EFNS** |  |  |  |  |  |  |
|  | Gauthier *et al* (2012) **CCCDTD4** |  |  |  |  |  |  |
|  | Gelenberg *et al.* (2010) **APA** |  |  |  |  |  |  |
|  | Dua *et al.* (2011) **WHO** |  |  |  |  |  |  |
|  | No Author (2012) **Availia-T** |  |  |  |  |  |  |
|  | Mitchell *et al.* (2013) **ICSI** |  |  |  |  |  |  |
