# Supplementary material for: Guidelines for dementia or Parkinson’s disease with depression or anxiety: a systematic review
Source: BMC Neurol. 2016 Nov 25;16:244. doi: 10.1186/s12883-016-0754-5 (PMC5124305; doi:10.1186/s12883-016-0754-5)
Supplement: Additional file 4: Table S2. — Mean Domain Question Scores From AGREE II Evaluation. (DOCX 16 kb) [file 12883_2016_754_MOESM4_ESM.docx]

**Table S2: Mean Domain Question Scores from AGREE II Evaluation**

| AGREE II Question | Mean Rating | SD |
| --- | --- | --- |
| Domain 1 - Scope and Purpose |  |  |
| Q1 - The overall objective(s) of the guideline is (are) specifically described. | 5.88 | 0.61 |
| Q2 - The health question(s) covered by the guideline is (are) specifically described. | 5.24 | 1.19 |
| Q3 - The population (patients, public, etc.) to whom the guideline is meant to apply is specifically described. | 4.96 | 0.20 |
| Domain 2 - Stakeholder Involvement |  |  |
| Q4 - The guideline development group includes individuals from all relevant professional groups. | 4.83 | 1.42 |
| Q5 - The views and preferences of the target population (patients, public, etc.) have been sought. | 3.25 | 1.92 |
| Q6 - The target users of the guideline are clearly defined. | 4.72 | 1.32 |
| Domain 3 - Rigour of Development |  |  |
| Q7 - Systematic methods were used to search for evidence. | 5.59 | 0.98 |
| Q8 - The criteria for selecting the evidence are clearly described. | 5.10 | 1.17 |
| Q9 - The strengths and limitations of the body of evidence are clearly described. | 5.34 | 0.65 |
| Q10 - The methods for formulating the recommendations are clearly described. | 4.93 | 1.21 |
| Q11 - The health benefits, side effects, and risks have been considered in formulating the recommendations. | 5.41 | 0.66 |
| Q12 - There is an explicit link between the recommendations and the supporting evidence. | 5.78 | 0.51 |
| Q13 - The guideline has been externally reviewed by experts prior to its publication. | 4.28 | 2.03 |
| Q14 - A procedure for updating the guideline is provided. | 3.16 | 1.73 |
| Domain 4 - Clarity of Presentation |  |  |
| Q15 - The recommendations are specific and unambiguous. | 5.50 | 0.79 |
| Q16 - The different options for management of the condition or health issue are clearly presented. | 5.63 | 0.65 |
| Q17 - Key recommendations are easily identifiable | 5.74 | 0.78 |
| Domain 5 - Applicability |  |  |
| Q18 - The guideline describes facilitators and barriers to its application. | 3.44 | 1.27 |
| Q19 - The guideline provides advice and/or tools on how the recommendations can be put into practice. | 3.72 | 1.53 |
| Q20 - The potential resource implications of applying the recommendations have been considered. | 3.37 | 1.46 |
| Q21 - The guideline presents monitoring and/or auditing criteria. | 3.50 | 1.43 |
| Domain 6 - Editorial Independence |  |  |
| Q22 - The views of the funding body have not influenced the content of the guideline. | 4.53 | 1.37 |
| Q23 - Competing interests of guideline development group members have been recorded and addressed. | 4.57 | 1.74 |
